# Plasmablastic lymphoma of the rectum: case report of a rare location and literature review

**DOI:** 10.1093/jscr/rjac030

**Published:** 2022-02-17

**Authors:** Razan Saleh AlMohamedi, Waleed AlRajban, Ammar AlRikabi, Maha Arafah

**Affiliations:** Department of Pathology, King Khaled University Hospital, Riyadh, Saudi Arabia; Department of Pathology, King Saud University, Riyadh, Saudi Arabia; Department of Pathology, King Khaled University Hospital, Riyadh, Saudi Arabia; Department of Pathology, King Saud University, Riyadh, Saudi Arabia; Department of Pathology, King Khaled University Hospital, Riyadh, Saudi Arabia; Department of Pathology, King Saud University, Riyadh, Saudi Arabia; Department of Pathology, King Khaled University Hospital, Riyadh, Saudi Arabia; Department of Pathology, King Saud University, Riyadh, Saudi Arabia

## Abstract

Plasmablastic lymphoma (PBL) is an aggressive and rare variant of diffuse large B-cell lymphoma, which is thought to occur in immunocompromised individuals, specifically HIV-positive patients. We report the case of a 27-year-old Saudi male with PBL. The patient had a low CD4 count at presentation, however, he was HIV negative at the time of diagnosis; also Human herpesvirus-8 was negative on immunohistochemical stain, but Epstein–Barr virus showed expression in scattered cells through the utilization of EBV-EBER.

## INTRODUCTION

Plasmablastic lymphoma (PBL) is an aggressive and a rare variant of diffuse large B-cell lymphoma (DLBCL), which is thought to occur in immunocompromised individuals, specifically HIV-positive patients. It is usually common in the oral cavity [1]. However, other anatomical sites that can be involved include the lung, spleen, gastrointestinal tract, liver, nasopharynx and the central nervous system [2, 3]. PBL follows a highly malignant behavior with a median survival rate between 5 and 11 months [1, 2].

## CASE REPORT

A 27-year-old male, otherwise healthy, is admitted electively in January 2021 for workup of a rectal mass. His symptoms started in October 2020 when the patient experienced symptoms of abdominal pain, distention, postprandial abdominal discomfort, mixture of mucus and blood in loose stool which was followed by constipation, as well as night sweats and unintentional weight loss of 25 kilograms over a period of 3 months. Family history was positive for acute myeloid leukemia affecting his brother who passed away during his adolescence. The patient underwent colonoscopy outside King Saud Medical City and found to have a mass in the rectum, which was diagnosed as DLBCL. Upon admission to our facility, he was subjected to further investigations, which included several rectal biopsies.

**Figure 1:**
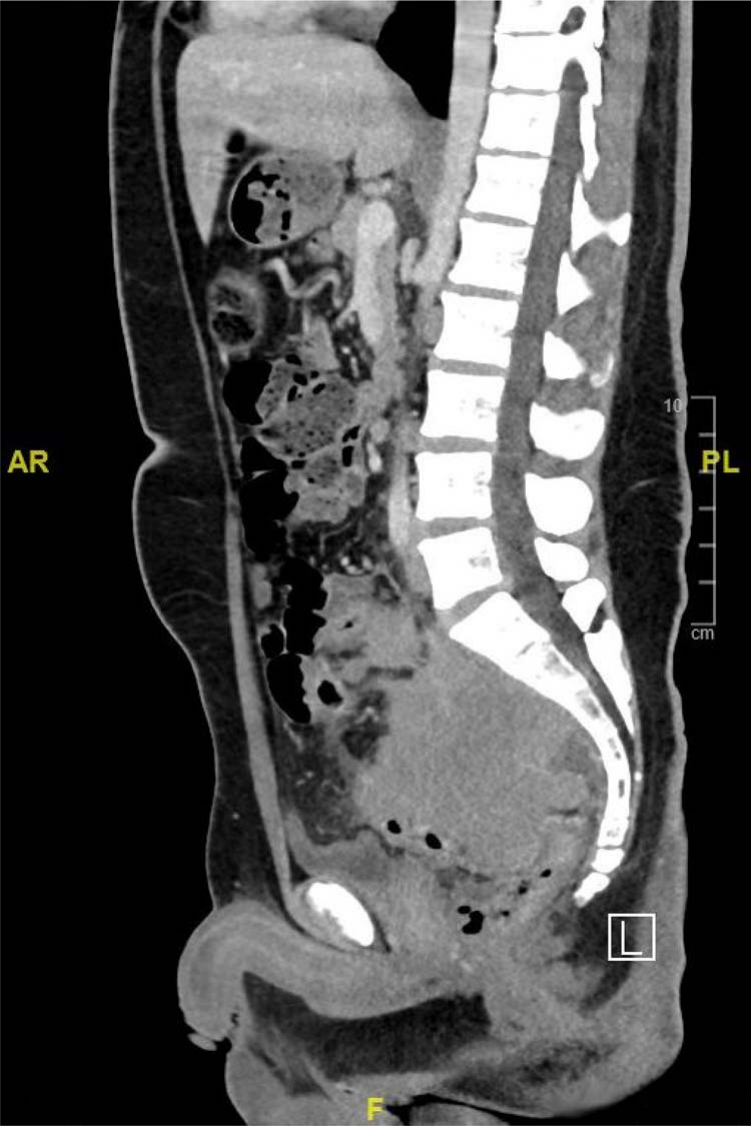
CT scan of the abdomen showed a large upper and mid-rectal mass.

Computed tomography (CT) scan of the abdomen showed a large upper and mid-rectal mass (Fig. 1). Colonoscopy was done and it revealed a polypoidal mass located 3 cm from the anal verge and extending up to 15 cm from anal verge. The mass was erythematous, and friable.

## HISTOLOPATHOLOGICAL FINDINGS

Two rectal biopsies were obtained from the patient in two different sittings; the first one was not conclusive and showed extensive necrotic tissue; however, it also demonstrated a highly suspicious malignant hematopoietic neoplastic process.

**Figure 2:**
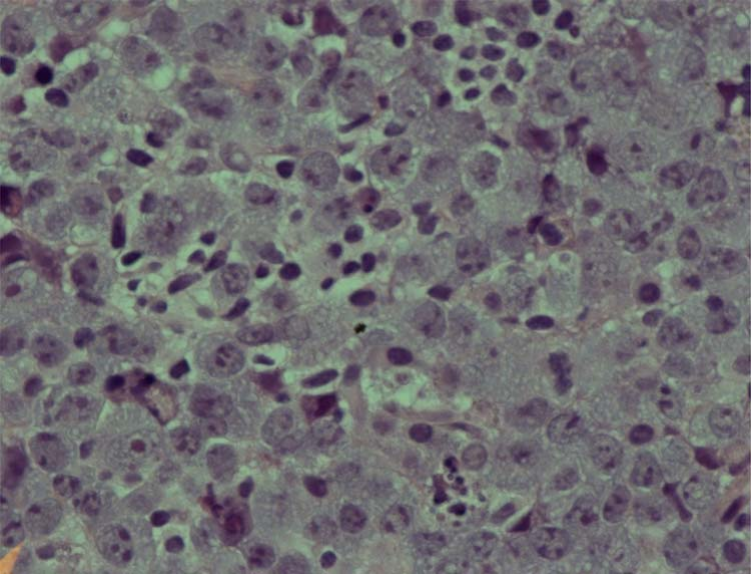
Photomicrograph showing an area of undifferentiated plasmablastic lymphoma; note the presence of large and pleomorphic tumor cells showing vesicular nuclei and conspicuous nucleoli with many apoptotic bodies: H/E stain X 600.

**Figure 3:**
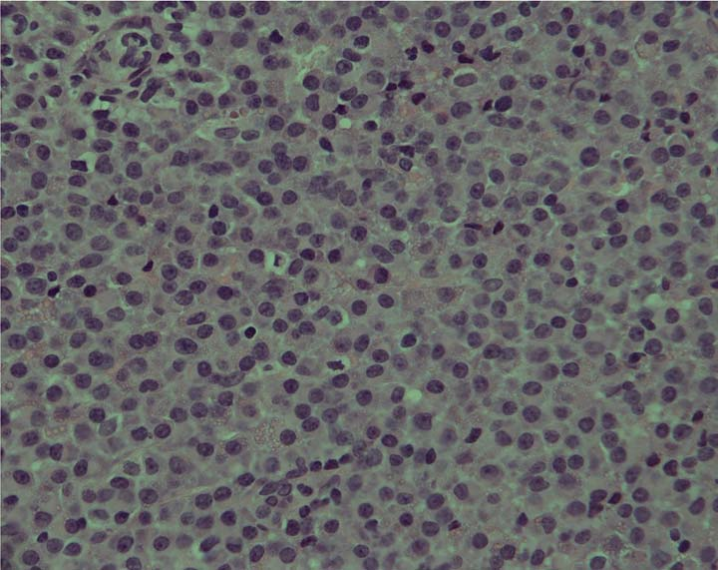
PBL; numerous neoplastic plasmacytoid cells with eccentric nuclei are seen; note the clear nuclear chromatin and conspicuous nucleoli in the majority of tumor cells: H/E stain X400B.

The second set of biopsies was straightforward and showed prominent involvement by malignant large lymphoid cells with large irregular nuclei, coarse chromatin, prominent nucleoli and moderate cytoplasm (Figs 2 and 3). Plasmacytoid differentiation is obvious. Increased mitosis and apoptosis were seen.

In addition, there were many foci showing sheets of low-grade plasmacytoid cells.

On immunostaining, the abnormal large cells were positive for CD38, MUM1, BOB1, OCT2, CD30 (focal) and EMA (focal) and negative for CD20, CD79a, PAX-5, CD138, CD56, HHV-8 and ALK-1. More importantly, there were scattered cells expressing EBV-EBER stain.

**Figure 4:**
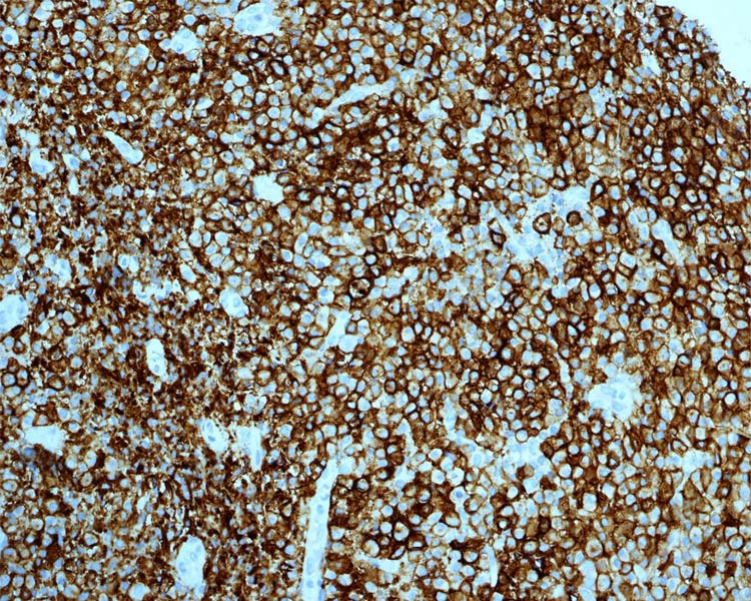
PBL; strong positive membranous staining with CD38 IHC stain X200.

**Figure 5:**
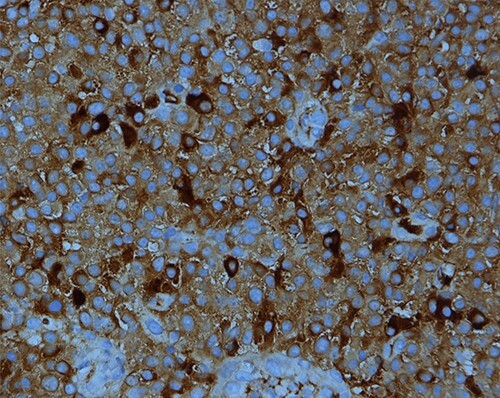
Kappa light chain restriction is obvious in this PBL; IHC stain for kappa light chain X400.

The low-grade plasmacytoid cells were positive for CD38, CD79a, CD20 (occasional cells) and MUM1 with Ki-67 expression of <10%. They also showed kappa light chain restriction (Figs 4 and 5).

The overall morphological and immunophenotypical findings are consistent with PBL. The presence of low-grade plasmacytoid component raised the possibility of preexisting marginal zone lymphoma with plasmacytoid differentiation, which has later on transformed to PBL.

Flow cytometry results showed CD4: 24% (low), CD8: 56% (elevated), CD19: 17% (Mild elevation), CD 4:CD 8 ratio 0.42% (decreased) and CD16:CD56 ratio 4% (elevated).

Bone marrow biopsy was performed, and it was negative for PBL.

## OUTCOME

The patient was shifted to the care of the hematology oncology team who started him on EPOCH (etoposide, prednisone, vincristine, cyclophosphamide and doxorubicin).

Unfortunately, the tumor metastasized to the central nervous system, and the patient expired 3 months after diagnosis.

## DISCUSSION

PBL was first described in 1997 as a very aggressive variant of DLBCL mainly presenting as lesions in extranodal sites and particularly the oral cavity. It is usually associated with immunodeficiency and Ebstein–Barr virus (EBV) infection. Although the pathogenesis is still uncertain, it is believed that PBL represents a neoplastic process stemming from post-germinal center B cells [1].

The diagnosis of PBL in immunosuppressed patients is well known; however, the Lysa group [2] has shown in their retrospective cohort study of 135 PBL cases that 45% of the patients diagnosed with PBL were neither immunosuppressed nor infected with human-immunodeficiency virus; when further analyzed, it was found that majority of them had a systemic inflammatory response or an autoimmune process.

PBL is known to have high association with EBV and human herpesvirus-8 (HHV8) [5]. Although our patient is HIV and HHV-8 negative and EBV positive, a recent study in Saudi Arabia demonstrated that all eight cases were negative for EBV; of these eight cases only one was positive for HHV-8 and two were positive for HIV. This raises the suspicion regarding the influence of geographic and genetic factors on the pathogenesis of PBL [6].

The plasmablastic morphology is characterized by diffuse large tumor cells with blastic differentiation and mitoses. These plamablastic tumor cells generally show the typical immunophenotype of plasma cells, expressing CD38, CD138 and MUM-1, and variably expressing CD79a, CD56, CD45, CD10, CD30 and EBER; the cells are negative for B-cell markers CD20 and PAX-5, with lambda light chain restriction [3].

In this case report, the large cells were positive for CD38. However, they were negative for CD138, CD20 and PAX5. The low-grade plasmacytoid cells were positive for CD38, CD79a, MUM-1 and CD20 in occasional cells, alongside a Ki-67 mitotic rate <10%. They also showed kappa light chain restriction. The overall morphological and immunological findings were consistent with PBL. The presence of low-grade plasmacytoid component raises the possibility of a preexisting marginal zone lymphoma with plasmacytoid differentiation, which was transformed to PBL. This raises the suspicion of other underlying causes of plasmablastic transformation in B-cell lymphomas.

Montes-Moreno et al. [4] have demonstrated that PBL is an age-related malignancy, affecting the patients with age-related immunodeficiency and young immunocompromised patients, in contrast to our case that represented a young patient who was firstly diagnosed with EBV-related DLBCL NOS.

## SUMMARY

In patients presenting with rectal lymphoproliferative disorders, the possibility of PBL should be considered if the morphologic features are suggestive. PBL can be seen in young patients who are HIV and HHV8 negative. PBL may occur as a result of transformation from other non-Hodgkin lymphomas. However, this point is in need of further investigations in the setting of larger studies.

## CONFLICT OF INTEREST STATEMENT

None declared.

## FUNDING

None.
